# Anatomy-Based Filler Injection: Treatment Techniques for Supraorbital Hollowness and Charming Roll

**DOI:** 10.3390/life15020304

**Published:** 2025-02-15

**Authors:** Gi-Woong Hong, Wonseok Choi, Jovian Wan, Song Eun Yoon, Carlos Bautzer, Lucas Basmage, Patricia Leite, Kyu-Ho Yi

**Affiliations:** 1 Samskin Plastic Surgery Clinic, Seoul 06577, Republic of Korea; 2 V Plastic Surgery, Daegu, Republic of Korea; 3 Medical Research Inc., Seoul, Republic of Korea; 4 Brandnew Aesthetic Surgery Clinic, Seoul, Republic of Korea; 5 Lifestyle Clinic, Sao Paulo, Brazil; 6 Synergie Clinic, Campo Grande, Brazil; 7 Patricia Leite Clinic, Belo Horizonte, Brazil; 8 Division in Anatomy and Developmental Biology, Department of Oral Biology, Human Identification Research Institute, BK21 FOUR Project, Yonsei University College of Dentistry, 50-1 Yonsei-ro, Seodaemun-gu, Seoul 03722, Republic of Korea; 9 You & I Clinic (Mokdong), Seoul 06001, Republic of Korea

**Keywords:** supraorbital hollowness, hyaluronic acid fillers, periorbital rejuvenation, eyebrow anatomy, pretarsal fullness

## Abstract

Supraorbital hollowness and pretarsal fullness, commonly known as the sunken eyelid and charming roll, respectively, are significant anatomical features that impact the aesthetic appearance of the periorbital region. Supraorbital hollowness is characterized by a recessed appearance of the upper eyelid, often attributed to genetic factors, aging, or surgical alterations, such as excessive fat removal during blepharoplasty. This condition is particularly prevalent among East Asians due to anatomical differences, such as weaker levator muscles and unique fat distribution patterns. Pretarsal fullness, also known as aegyo-sal, enhances the youthful and expressive appearance of the lower eyelid, forming a roll above the lash line that is considered aesthetically desirable in East Asian culture. Anatomical-based filler injection techniques are critical for correcting these features, involving precise placement within the correct tissue planes to avoid complications and achieve natural-looking results. This approach not only improves the aesthetic appeal of the eyelid but also enhances the overall facial harmony, emphasizing the importance of tailored procedures based on individual anatomy and cultural preferences.

## 1. Introduction

Supraorbital hollowness, commonly referred to as sunken eyelid, is a condition characterized by a recessed appearance of the upper eyelid that imparts a fatigued, aged, or sleepy look. This appearance can result from congenital factors, natural aging processes, or procedures such as blepharoplasty that involve the excessive removal of orbital fat [1,2,3,4,5,6,7]. Individuals, particularly those of East Asian descent, are more prone to this condition due to anatomical differences, including a generally weaker levator palpebrae superioris muscle that often lacks attachment to the dermal tissue. As a result, the eyelid does not fully retract when the eyes are open, contributing to the appearance of thickened skin and a lack of double eyelid formation [8]. Age-related changes further exacerbate the condition as fat beneath the orbital septum diminishes, leading to a more pronounced hollow look and sagging skin [9]. This effect, combined with the structural characteristics of the upper eyelid and surrounding areas, results in the distinctive sunken appearance associated with supraorbital hollowness.

Flat eyebrows, often seen in conjunction with supraorbital hollowness, can significantly impact the overall facial aesthetic. The position, shape, and volume of the eyebrows play a crucial role in defining facial expressions and impressions. In many cases, East Asians experience a greater accumulation of subcutaneous fat in the upper eyelid and brow region, contributing to a swollen appearance that becomes more pronounced with age. As the eyebrows lose volume and descend over time, they can further accentuate the hollow look of the supraorbital area. Proper pre-treatment considerations, including the evaluation of the patient’s facial structure, anatomy, and aesthetic goals, are essential to developing an effective treatment plan. By addressing these factors, enhancing the hollow areas along the orbital rim and adjusting the eyebrow shape, a more youthful and alert appearance can be achieved, ultimately improving the overall aesthetic harmony of the eyes and face.

The popularity of pretarsal roll or charming roll fillers, especially among Koreans, stems from cultural preferences for a youthful, expressive eye appearance. Known as “aegyo-sal”, the enhanced lower eyelid roll is seen as adding charm and a fresh, lively look, which aligns with East Asian beauty ideals that favor youthful, natural features. In this context, pretarsal roll fillers play a central role in achieving this aesthetic, with many opting for subtle filler techniques that emphasize this feature without compromising the natural eyelid contour.

The primary objective of this study is to provide an anatomy-based approach to filler injection techniques in the periorbital region, focusing on the treatment of supraorbital hollowness and enhancement of pretarsal fullness, or “aegyo-sal”. This review aims to address the unique anatomical considerations required for achieving natural and balanced aesthetic outcomes, particularly in East Asian populations, who may present with specific anatomical characteristics such as distinct fat distribution patterns and muscle attachments. The hypothesis driving this review is that precision-based filler injections, tailored to anatomical planes within the periorbital area, can enhance aesthetic outcomes by providing effective, natural-looking volume restoration while minimizing risks of complications such as vascular occlusion, bruising, and filler migration. Through a structured review of existing literature and anatomical analysis, this study seeks to provide clinicians with practical, evidence-based recommendations to guide safe and effective periorbital filler procedures.

## 2. Materials and Methods

Keywords including “Supraorbital Hollowness”, “Sunken Eyelid”, “Pretarsal Fullness”, “Charming Roll”, “Hyaluronic Acid Fillers”, “Eyebrow Rejuvenation”, and “Periorbital Rejuvenation” were searched in the MEDLINE, PubMed, and Ovid databases for relevant studies published on anatomical insights, clinical approaches, and treatment techniques specific to the periorbital region. This search yielded a total of 182 studies. Studies were further reviewed based on criteria such as detailed descriptions of injection techniques, anatomical dissections, and specific outcomes in periorbital rejuvenation, especially among East Asian populations. After applying these criteria, 150 studies were excluded due to lack of detailed methodology, limited sample size, or the absence of objective outcome measurements, resulting in a final selection of 32 studies. All studies were then classified according to the Oxford Centre for Evidence-Based Medicine evidence hierarchy.

## 3. Supraorbital Hollowness (Sunken Eyelid) and Flat Eyebrow

### 3.1. Pre-Treatment Considerations

Supraorbital hollowness, often termed sunken eyelid, is characterized by a recessed appearance of the upper eyelid, either due to congenital factors or the natural aging process [10]. This condition imparts a fatigued, sleepy, or aged look [11]. Contributing factors include genetic predisposition, age-related loss of eyelid fat, or the excessive removal of orbital fat during blepharoplasty [12,13,14]. In particular, East Asians may experience this condition more prominently due to anatomical differences [15]. The levator palpebrae superioris muscle, which elevates the upper eyelid by attaching to the tarsal plate, is generally weaker in this population and frequently lacks attachment to the dermal tissue. Consequently, the upper eyelid does not retract fully when the eyes are open, preventing the formation of a double eyelid and resulting in the appearance of sagging and thickened skin [16,17].

East Asians commonly exhibit a greater accumulation of subcutaneous fat in the upper eyelid and the surrounding eyebrow region. As this fat descends, it contributes to a swollen and puffy appearance of the upper eyelid [18]. With advancing age, the outer portion of the eyelid, particularly outside the tarsal plate, may experience sagging and become more pronounced, while the area around the orbital margin may appear hollow due to the reduction in fat beneath the orbital septum. This progressive weakening of the eyelid muscles with age results in senile ptosis and increased skin sagging, leading to a characteristic sunken appearance of the eyelid, which can look hollow and fatigued [17].

**Figure 1:**
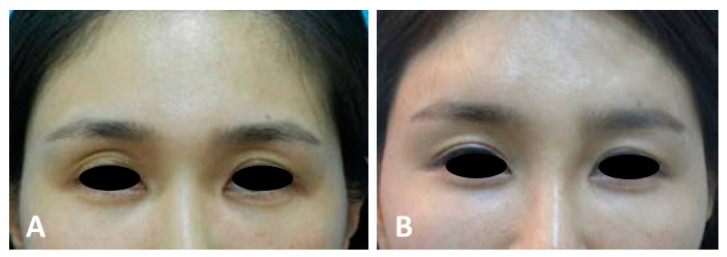
Before (**A**) and after (**B**) treatment of supraorbital hollowness.

A sunken eyelid often conveys a tired and sleepy appearance, with the eyes lacking sharpness, resembling ptosis [11]. This condition typically results in the development of a prominent supratarsal lid crease, which appears along the orbital margin and is situated significantly higher than the upper margin of the tarsal plate, where the typical double eyelid crease forms. When such a pronounced orbital lid crease develops, the skin tends to fold inward at this elevated crease rather than at the double eyelid line below, causing the lower crease to become less defined and blurred. In these instances, enhancing the hollow areas along the orbital rim can improve the tired and sleepy appearance. This procedure also helps to soften the pronounced orbital lid crease, thereby making the lower double eyelid crease more defined and prominent. As a result, the overall appearance of the eyes becomes sharper and more alert (Figure 1) [19].

### 3.2. Procedure for Treating Supraorbital Hollowness

When addressing supraorbital hollowness, it is essential to perform the procedure with the patient in an upright position and their eyes open. The technique should involve retrograde linear threading and tiny, slow injections using a small-particle, soft-consistency hyaluronic acid (HA) filler, which allows for effective molding. Achieving optimal results and minimizing risks depends critically on selecting the appropriate injection plane [1].

Injecting within the orbicularis oculi muscle is generally discouraged due to the muscle’s thinness, which complicates precise injections and increases the risk of hemorrhage owing to the muscle’s rich vascular supply [20]. Additionally, although the loss of fat within the orbital septum may lead practitioners to consider injecting directly into the orbital fat, this approach is not recommended. Managing hemostasis in this region is difficult if bleeding occurs, and the formation of a hematoma within the septum could damage the septum itself, potentially compromising its role as a lubricant during eyelid movement.

**Figure 2:**
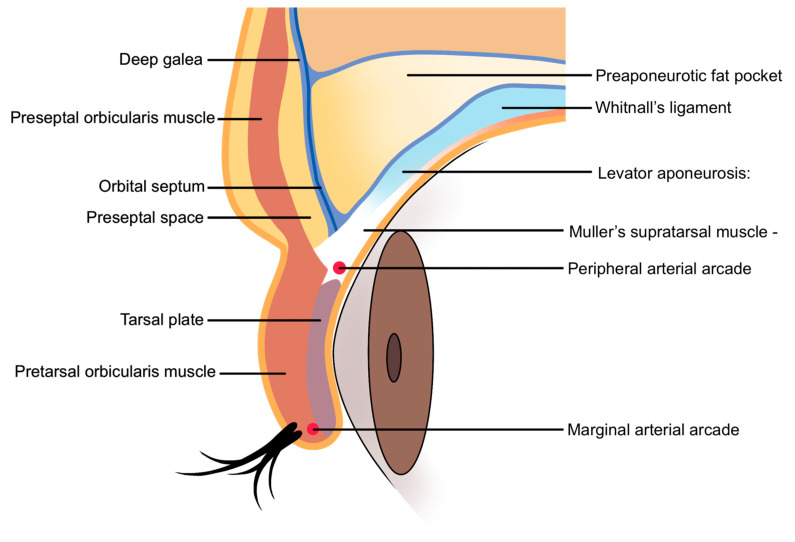
Anatomical layers of the supraorbital region.

The preferred injection plane for treating supraorbital hollowness is the deep fat layer located beneath the orbicularis oculi muscle, following the margin of the orbital rim. This layer is continuous with the retro-orbicularis oculi fat (ROOF) pad in the eyebrow region. However, in cases of sunken eyelids, the deep fat layer within the eyelid is often minimal or absent. Consequently, the filler should be injected into the preseptal space, which lies beneath the orbicularis oculi muscle but outside the orbital septum (Figure 2) [21].

**Figure 3:**
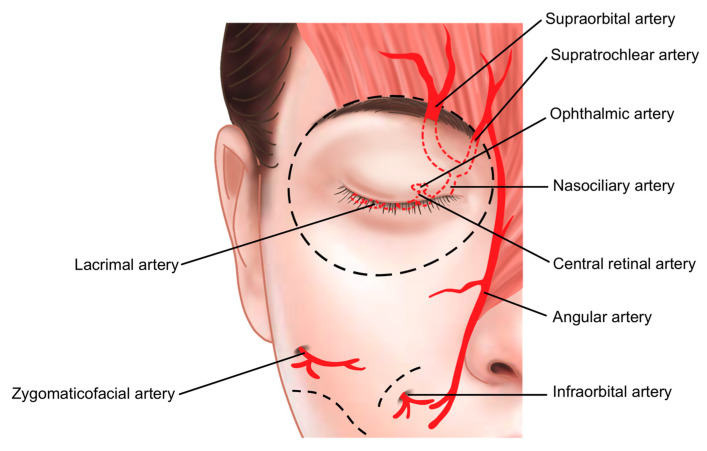
Vascular structures of the orbital region.

The medial portion of the orbital rim contains critical vascular structures, including the supratrochlear and supraorbital arteries, both branches of the ophthalmic artery supplying the forehead and glabella regions. These vessels connect to the central retinal artery within the orbit, necessitating extreme caution during procedures in this area. To minimize the risk of complications such as intra-arterial injection and other forms of vascular compromise, it is recommended to use a cannula instead of a needle (Figure 3).

**Figure 4:**
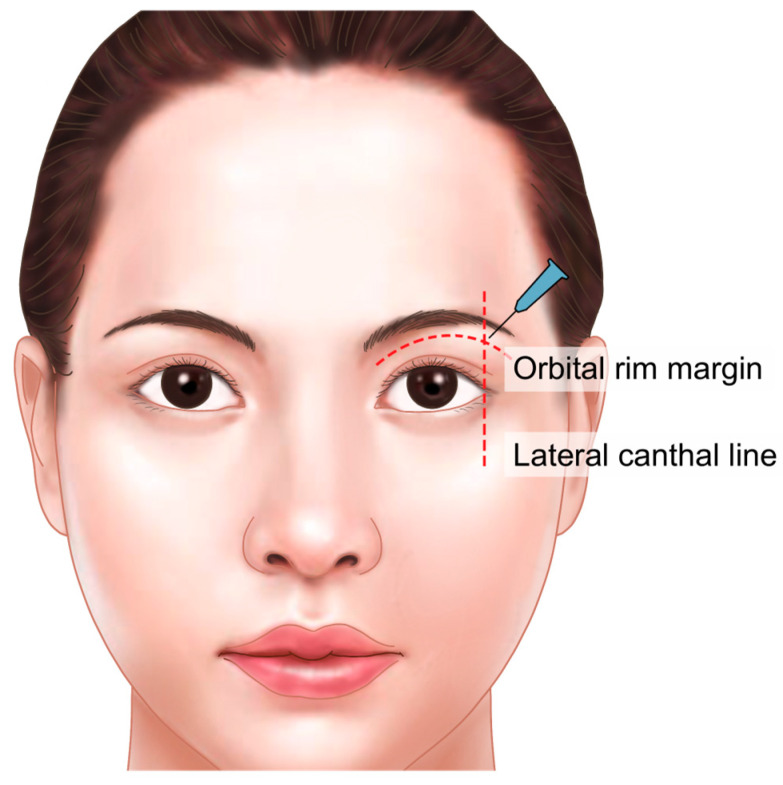
Injection entry point and technique for the cannula. Injection entry point: Vertical line drawn above or outside the lateral canthus, around the lower margin of the superior orbital rim. Focus on the medial and middle parts of the periorbital rim, under the brow, to avoid the supraorbital and supratrochlear main arteries. Above the supratarsal lid crease and below the orbicularis retaining ligament. Injection technique: Patient in vertical sitting position with voluntarily opened eyes. Retrograde linear tiny injection technique with very slow release.

The puncture point for cannula insertion may vary slightly depending on the extent of the hollow area, but it is generally located where a vertical line drawn from the lateral canthus intersects with the orbital rim. To ensure accurate placement, the entry point should be on the skin overlying the firm orbital rim, rather than on the looser skin below the rim. After insertion, the cannula should pass through the fibrous tissue of the orbicularis retaining ligament, part of the muscle layer. Once through this tissue, a slightly looser space beneath it can be felt. For further confirmation, the cannula should be advanced until it touches the bone of the orbital rim, then withdrawn slightly to position it correctly in the space just beneath the orbicularis oculi muscle (Figure 4).

**Figure 5:**
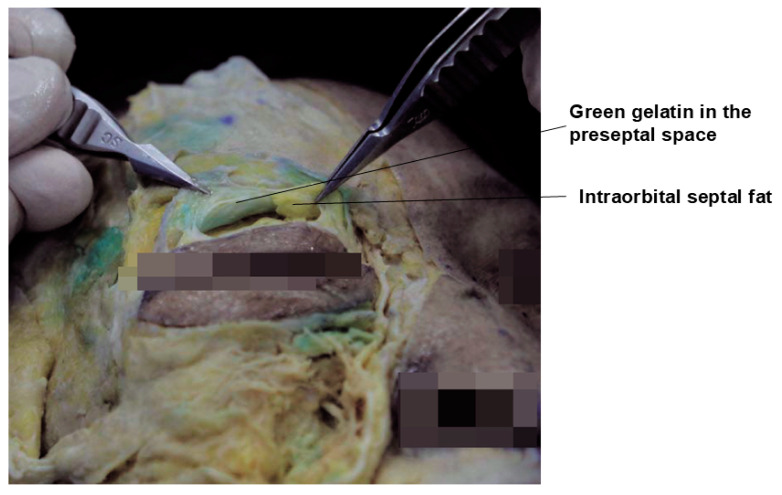
Anatomy of the preseptal space.

Next, the cannula should be advanced along the hollow groove of the orbital rim, maintaining a consistent depth throughout the procedure. It is critical to ensure that the cannula tip reaches the desired location while preserving the initial depth. If fibrous tissue is encountered, gently moving the cannula back and forth can create space and facilitate smooth advancement. Once the target position is reached, the cannula should be slowly withdrawn while carefully injecting small amounts of HA filler. This method minimizes the risk of creating an uneven surface. As previously mentioned, since there is little to no fat layer beneath the orbicularis oculi muscle, the cannula should remain in the preseptal space, outside the orbital septum (Figure 5).

During the procedure, it is crucial to continuously monitor the depth and volume of the filler to avoid injecting too superficially or excessively, as this can result in noticeable lumpiness or bulging when the patient closes their eyes. Overcorrection can cause the eyes to appear swollen or lead to filler migration to the lower eyelid. Therefore, it is safer to perform a moderate correction and evaluate the results before making further adjustments.

**Figure 6:**
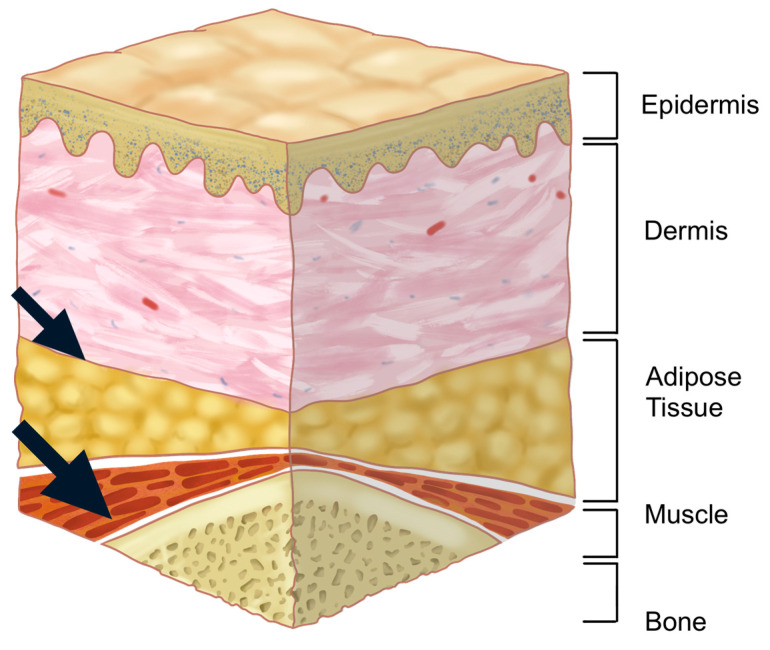
Injection planes: Supraperiosteal and submuscular injections around the orbital rim over the orbital septum to fill the hollowness. Subdermal injection of very soft HA filler to smooth the surface and remove unnecessary multiple eyelid lines.

After achieving the desired correction, if areas appear underfilled or if the contours are not smooth, a small amount of very soft HA filler can be injected into the subdermal layer to refine the surface. Given the thinness of the eyelid skin, care must be taken to prevent the formation of beads or nodules during this final step (Figure 6).

Patients with severe ptosis or significant ocular protrusion may not achieve favorable outcomes from this procedure, and their symptoms could worsen, making treatment inadvisable in such cases. Additionally, the presence of scar tissue from previous surgeries or trauma may complicate even filler distribution, and this should be carefully considered during treatment planning.

### 3.3. Procedure for Treating Flat Eyebrows

The position, shape, and volume of the eyebrows, situated just below the forehead, play a critical role in shaping a person’s overall facial impression. As individuals age, the volume in the eyebrow region diminishes, leading to a downward shift in the eyebrows [5,22]. This phenomenon is more prominent in Western populations compared to East Asian populations, a difference attributable to variations in bone structure and skin thickness [23].

Eyebrows, traditionally understood as the arch-shaped hair along the raised area of the supraorbital ridge—the bony prominence above the eye socket—typically measure between 7 to 11 mm in width and approximately 5 to 6 cm in length [24]. The growth pattern and shape of the eyebrows are influenced by factors such as race, gender, and age [25]. Unlike scalp hair, eyebrow hair has a shorter growth phase, which results in its relatively shorter length [26].

The skin in the eyebrow area is governed by several facial muscles, including the frontalis muscle, orbicularis oculi muscle, corrugator supercilii muscle, and procerus muscle. These muscles facilitate eyebrow movement during facial expressions, thereby enhancing facial expressiveness and enabling effective communication [27].

The primary functional role of eyebrows is ocular protection. They prevent rain, snow, and sweat from directly entering the eyes. Additionally, the raised orbital bone beneath the eyebrows acts as a barrier against dust and small particles, while also providing shade to protect the eyes from sunlight and bright light [28]. Beyond their protective function, the movement of the eyebrows during facial expressions significantly influences a person’s overall facial expression, conveying emotions such as surprise or anger. The shape of the eyebrows is a key determinant of a person’s appearance. In East Asian physiognomy, the shape of the eyebrows is believed to reflect an individual’s personality, image, and even fortune, leading to an increasing interest among men in eyebrow grooming and shaping [29,30,31].

Although the loss of volume and subsequent drooping of the eyebrows due to aging is generally less pronounced in East Asians, Western individuals often place greater emphasis on the position and volume of the eyebrows, considering them crucial to achieving a softer facial expression [32].

### 3.4. Optimal Eyebrow Position and Shape

**Figure 7:**
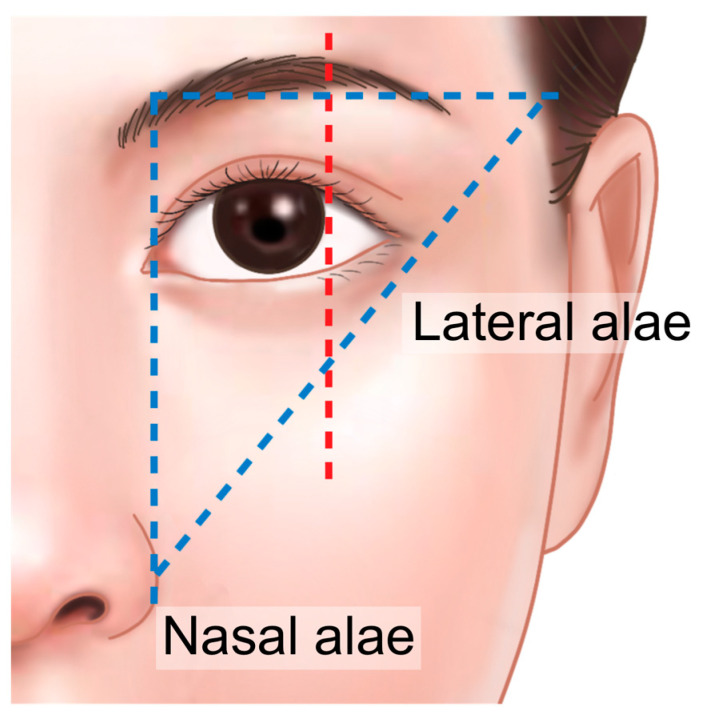
Ideal position and shape of the eyebrow.

The ideal position and shape of the eyebrows are typically determined by specific facial landmarks. The inner end of the eyebrow should align vertically with the lateral margin of the nasal alae, while the outer end should align with a line drawn from the lateral margin of the nasal alae through the lateral canthus of the eye. The highest point of the eyebrow, usually located at the lateral third, should be positioned along a vertical line drawn from the lateral limbus of the eye (Figure 7) [24,33,34].

**Figure 8:**
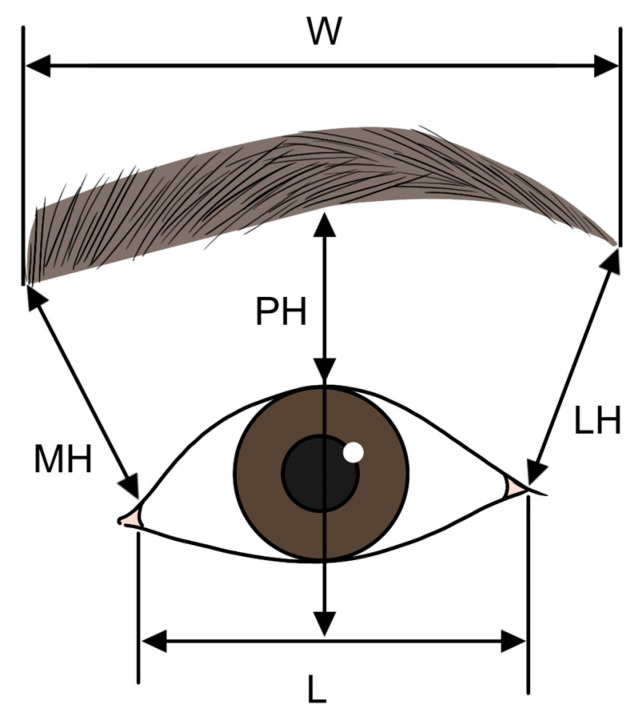
Ratio difference between the size of the eye and eyebrow.

Considering the proportional relationship between the eyes and eyebrows, when the width of the eye (L) is set as 1 unit, the straight length of the eyebrow (W) should ideally measure approximately 1.63 units. The distance from the medial canthus of the eye to the inner end of the eyebrow (MH) should be around 0.53 units, while the distance from the lateral canthus to the outer end of the eyebrow (LH) should be about 0.60 units. The distance from the center of the pupil to the eyebrow (PH) should be approximately 0.36 units (Figure 8) [35].

However, it is important to recognize that eyebrow shapes vary according to ethnicity, culture, and fashion trends. In Korea, for instance, eyebrow tattooing has become highly popular, with reports suggesting that nearly all older women engage in this practice, typically favoring straight eyebrows. Despite such trends, it is crucial to acknowledge that the most suitable eyebrow shape may vary depending on the individual’s facial structure [36].

**Figure 9:**
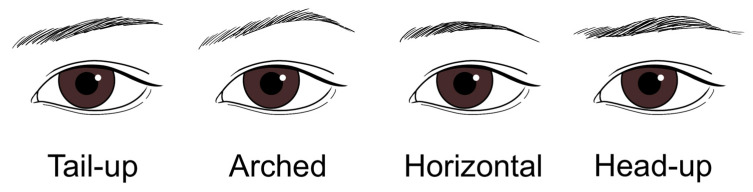
Common classification of eyebrow shapes around the world.

Globally, eyebrow shapes can be broadly categorized into four common types: arched, head-up, tail-up, and horizontal (Figure 9) [37]. While straight eyebrows have become a popular trend in Korea, with many considering this to be the preferred shape among Koreans, surveys indicate that over half of Koreans favor a naturally arched eyebrow shape [38]. A notable difference in preference between Eastern and Western cultures is that while East Asians often perceive the tail-up eyebrow shape as overly harsh and therefore less desirable, many Westerners favor this shape as much as they do the arched style. This difference in preference is likely influenced by the fundamental differences in facial structure between Eastern and Western populations, as well as cultural tendencies in the West to embrace more prominent and defined features.

Regardless of personal preferences, it is advisable for individuals to select an eyebrow shape that complements their facial features. Healthcare providers should consider this harmony when recommending eyebrow shapes. The current trend toward straight eyebrows, driven by the popularity of eyebrow tattoos, often overlooks the fact that the most suitable eyebrow shape varies depending on face shape. Generally, for a round face, somewhat angular, short, and high eyebrows are more flattering; for a square face, rounded arched eyebrows are ideal; and for longer faces, a more horizontal, straight eyebrow shape is considered more appropriate.

**Figure 10:**
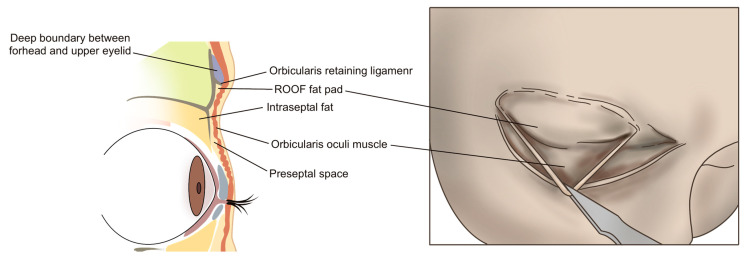
Retro-orbicularis oculi fat (ROOF) in the eyebrow region.

As individuals age, the retro-orbicularis oculi fat (ROOF) layer beneath the orbicularis oculi muscle, which provides volume to the eyebrow area, may diminish, altering the shape of the eyebrow–lid complex and causing the eyebrows to appear droopy. Restoring volume to the ROOF can tent and lift the eyebrows, helping to restore their original fullness and potentially elevating the outer portion of the eyebrows as well (Figure 10) [39,40].

**Figure 11:**
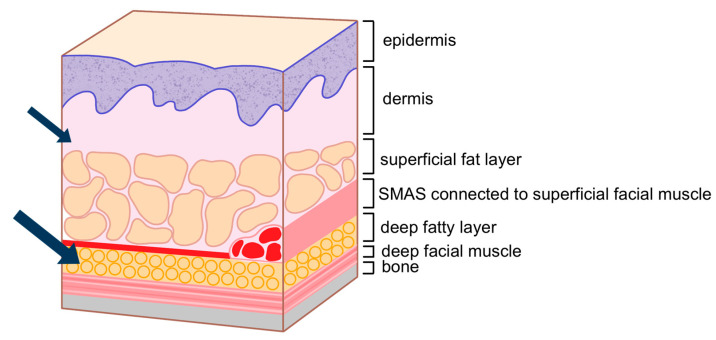
Injection plane for the cannula. Submuscular injection into ROOF (retro-orbicularis oculi fat) for eyebrow augmentation. Subdermal injection of very soft filler to even out the surface and remove unnecessary multiple eyelid lines.

Typically, the area medial to the midpupillary line is composed of firm skin tissue that is well integrated with the underlying muscle, meaning it does not usually experience significant sagging or volume loss. Therefore, the focus should be on restoring volume in the area lateral to the midpupillary line. After making a needle puncture at the lateral eyebrow end, a cannula should be inserted into the ROOF fat pad beneath the orbicularis oculi muscle. Using a moderately viscous HA filler, volume should be appropriately enhanced. Subsequently, a softer filler should be injected into the dermis and subdermal layers to ensure a smooth surface finish (Figure 11) [41].

## 4. Pretarsal Fullness/Lower Eyelid Charming Roll

### 4.1. Pre-Treatment Considerations

**Figure 12:**
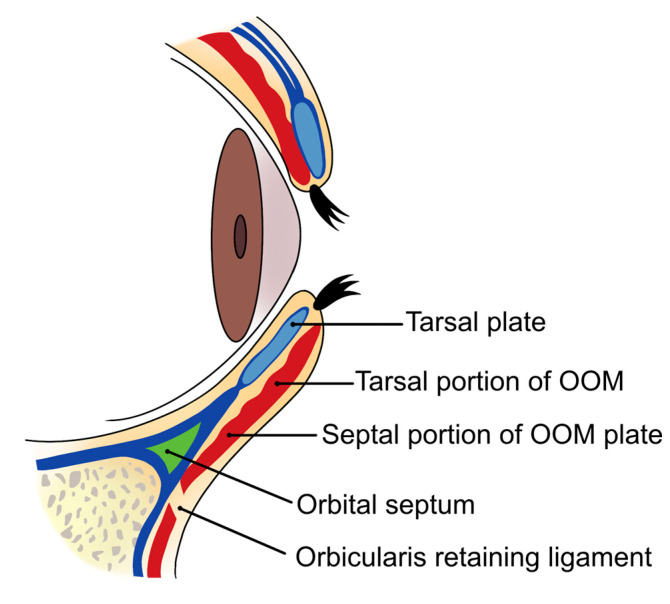
Structure of the lower eyelid roll muscle.

The pretarsal margin of the lower eyelid, where the tarsal plate is located, measures approximately 2 mm in thickness. The inner portion of the eyelid is angular, facilitating the close adherence of the tarsal plate to the eyeball. In contrast, the outer portion, which houses the cilia (eyelashes), is rounder and thicker, a feature often referred to as the “charming roll” or “aegyo-sal” (Figure 12) [42,43].

Pretarsal fullness, characterized by the protrusion of the orbicularis oculi muscle in the tarsal region anterior to the tarsal plate, creates a roll-like appearance above the subtarsal line, particularly evident when smiling. Notably, this area has minimal or no subcutaneous fat both above and below the orbicularis oculi muscle. A prominent aegyo-sal is associated with a youthful and charming appearance, making the eyes appear larger—a desirable attribute in East Asian aesthetics [41]. In contrast, Western aesthetics typically do not favor pronounced aegyo-sal, as excessive fullness can make the eyes appear smaller; therefore, a more moderate size and shape are preferred.

For optimal aesthetic outcomes, the aegyo-sal should be symmetrical on both sides, not droopy, and present as a continuous volume extending naturally from the inner to the outer corner of the lower eyelid. The fullness should be positioned as close to the lash line as possible. Typically, the aegyo-sal is not visible when the face is at rest but becomes prominent when smiling. Ideal candidates for this procedure are those whose lower eyelid muscles naturally form the aegyo-sal shape when the skin beneath the lower eyelid is gently pushed upward, indicating good skin elasticity and minimal sagging.

In youth, the creation of an aegyo-sal enhances a youthful and endearing appearance by mimicking the effect of a smiling expression. However, as one ages, the skin beneath the eyes tends to sag, and the underlying muscles may lose tone, resulting in a reduction in the muscle volume that supports the aegyo-sal. This leads to a flatter, more tired, and sullen appearance. In such cases, restoring the volume of the aegyo-sal through treatment is necessary to rejuvenate the area. Additionally, augmenting the aegyo-sal can also improve the appearance of dark circles and reduce fine wrinkles due to the skin expansion effect.

When injecting fillers into the aegyo-sal area, it is recommended that the patient smile first to identify the natural crease formed by the contraction of the lower eyelid muscle. Care must be taken to avoid adding volume below this line. Typically, a width of 4–6 mm from the subciliary line is appropriate. The filler can be administered to create a consistent volume along the entire lower eyelid, resembling a roll cake, or the volume can be strategically varied, with the central portion being the fullest, followed by a slightly less full outer portion, and the least volume in the inner portion. Some patients may prefer a slightly thicker appearance towards the lateral third of the eyelid.

The shape and thickness of the aegyo-sal can be customized to the patient’s preferences. The procedure can be performed using either a needle or a cannula, although a needle is generally preferred for creating a more precise shape. The challenges of this procedure include a high likelihood of bruising, the potential for an uneven surface, and the risk of an unnatural appearance if the area is overfilled. Additionally, because the filler must be administered in small, controlled amounts, there is a tendency for the aegyo-sal to appear discontinuous, with gaps or breaks in the contour when viewed as a whole. While the use of a cannula can mitigate some of these concerns, its manipulation can be challenging, which is why most practitioners opt for needle-based injections.

### 4.2. Procedure Method

Aegyo-sal enhancement is recognized as a procedure that can cause considerable discomfort. Consequently, it is recommended to apply a generous amount of topical anesthetic ointment to the lower eyelid, covering the area with a wrap for at least 10–20 min, or alternatively, to perform an infraorbital nerve block prior to the procedure. When utilizing a needle, it is advisable to opt for a short needle, and typically, a maximum of 0.2–0.4 mL of filler is injected per side. The practitioner should carefully evaluate the differences in appearance between a neutral facial expression and a smile to determine the precise locations and amounts of filler required.

To prevent an uneven or overly prominent appearance, it is preferable to use a soft HA filler rather than a firm one. Injecting the filler too deeply may result in general puffiness without achieving the desired plumpness, while injecting too superficially may cause surface irregularities, such as discontinuous lines or localized protrusions. Moreover, the filler may exhibit a bluish tint through the skin, known as the Tyndall effect. If the Tyndall effect is pronounced, even molding may not correct it, necessitating the dissolution of the filler and a subsequent attempt at the procedure.

**Figure 13:**
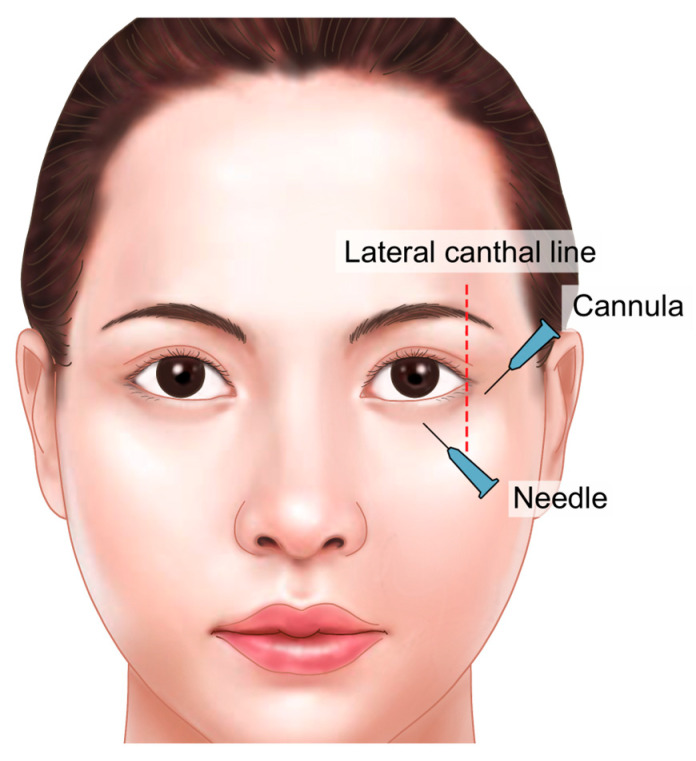
Injection techniques for the cannula or needle. Linear threading, retrograde tiny injection, very slow release, serial puncture, and tenting technique.

When using a needle, the filler is injected directly into the targeted area of the aegyo-sal. If using a cannula, a puncture point is created a few millimeters outside the lateral canthus, and the cannula is inserted following the needle puncture. The procedure is typically performed in stages, beginning with the central portion, followed by the lateral and medial areas. The filler is injected gradually and gently, employing techniques such as retrograde tiny injection, linear threading, and tenting to achieve a smooth and natural-looking volume enhancement (Supplement Video S1, Figure 13, and Table 1).

**Figure 14:**
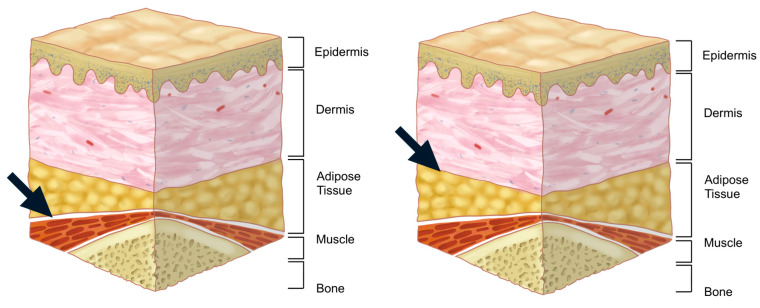
Injection planes: Deep subdermal or supramuscular injections. Subdermal injection to smooth the surface, close to the eyelash.

The author favors a dual-plane technique for achieving the desired pretarsal fullness (aegyo-sal). This method involves initially injecting filler beneath the orbicularis oculi muscle or within the superficial layer of the muscle to establish the overall shape. After the primary contour is formed, any areas that appear deficient or lack sufficient volume are further refined by injecting additional filler into the dermal or subdermal layers to smooth the surface and ensure a natural appearance (Figure 14).

When using a cannula for this procedure, the entry point should be positioned along the outer edge of the desired pretarsal fullness, as close to the lower eyelashes as possible, to avoid creating an unnatural appearance lower on the eyelid. The cannula is then inserted, keeping it close to the lower eyelashes and advancing it within the muscle or a slightly shallower layer. Once the cannula tip reaches the target area, filler is slowly injected while maintaining consistent pressure, gradually retracting the cannula to ensure an even distribution and uniform shape of the aegyo-sal.

For needle injections, the area is typically divided into three sections, with filler injected into each section to create the overall shape, followed by the addition of small amounts to any areas that still appear lacking. Even when dividing the area for injections, it is crucial to maintain a consistent depth throughout the procedure. The filler should be injected as close to the lower eyelid margin as possible, and the injection plane should be within or just below the orbicularis oculi muscle to minimize the risk of damaging the inferior palpebral arterial arch.

Inserting the needle too deeply can pose a risk of injuring the inferior palpebral arterial arch, which runs beneath the orbicularis oculi muscle, and potentially injecting filler directly into this vessel. This significantly increases the risk of complications. Therefore, maintaining a consistent depth during the procedure is essential to ensure the needle tip does not penetrate below the muscle layer.

The inferior palpebral arterial arch is formed by the anastomosis of the medial palpebral artery, which branches from the supratrochlear artery on the medial side of the orbit, and the lateral palpebral artery, which branches from the lacrimal artery on the lateral side. These arteries are components of the superior and inferior palpebral arterial arches. The significance of this vascular structure lies in its origin: the supratrochlear and lacrimal arteries are branches of the ophthalmic artery, which itself branches from the internal carotid artery. If filler is inadvertently injected into the inferior palpebral artery, there is a risk of retrograde flow, which could allow the filler to travel through these vessels and reach the central retinal artery, potentially leading to blindness [44].

**Figure 15:**
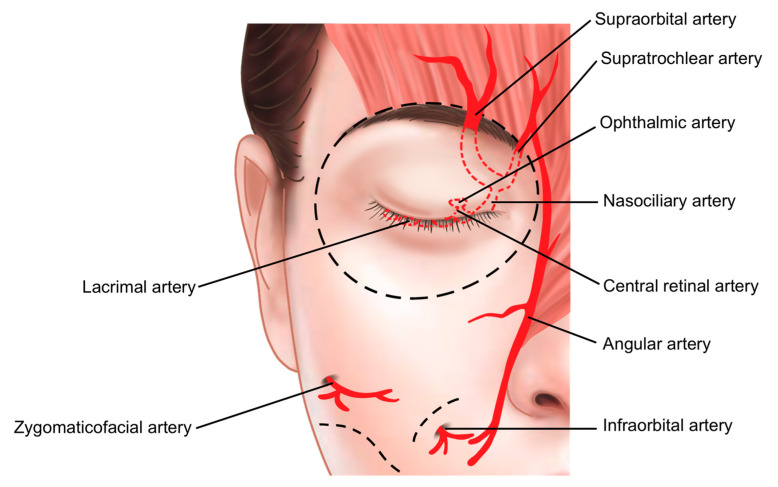
Anatomy of the superior and inferior palpebral arteries.

Given that the inferior palpebral artery is located deep beneath the orbicularis oculi muscle, injecting the filler within or slightly superficial to the muscle can help minimize the risk of vascular complications. Additionally, injecting as close to the lower eyelid margin as possible, considering the pathway of these vessels, enhances the safety of the procedure (Figure 15).

During the procedure, it is critical to inject the filler as close to the eyelashes as possible to avoid an excessively thickened appearance below the eyelashes, which would leave the area near the lashes too hollow. To prevent the filler from migrating downward, the non-dominant hand should support the tissue beneath the injection site. It is also important to assess the presence and degree of bulging orbital fat under the eyes before the procedure, as this can cause the lower eyelid and filler-enhanced area to appear excessively swollen when the patient smiles.

Filler injection for enhancing pretarsal fullness (aegyo-sal) is not recommended in certain cases. These include situations where the eyelid roll muscle is underdeveloped, the eye fissure (palpebral fissure) is either too wide or too narrow, the outer corners of the eyes are significantly elevated or lowered, the eyes are excessively sunken or protruding, the lower eyelid skin is too thick, there is severe darkening under the eyes, significant skin laxity is present, or there is pronounced bulging of the lower eyelid fat. Additionally, patients with scars from previous lower eyelid surgeries may encounter challenges with this procedure, making it difficult to achieve a natural-looking result.

For patients who have undergone lower eyelid surgery, creating pretarsal fullness can enhance their appearance, but it requires careful consideration due to potential scarring. If scarring has caused the eyelashes to invert or become misaligned, it is advisable to avoid the procedure altogether. In cases where scarring is not severe, it is possible to proceed by injecting the filler close to the eyelashes. However, starting with a deep injection can cause the filler to bypass the scarred area, leading to an uneven result where the scarred region remains flat. Therefore, it is recommended to begin with a shallow injection, gradually increasing the depth based on the observed contour, to achieve a balanced and natural appearance.

In patients who have undergone lower eyelid surgery, the initial filler injection may result in an uneven appearance due to adhesions caused by surgical scars, leading to irregular filler distribution. However, over the course of 1–2 weeks, as the patient naturally opens and closes their eyes, the filler typically disperses and settles into a more uniform contour. If the irregularities persist after this period, minor depressions can be improved by using a fine needle to inject a very soft, small-particle filler, which can help smooth out the surface and achieve a more even appearance.

## 5. Results

In this study, we identified key anatomical layers within the supraorbital and pretarsal regions essential for achieving natural, safe filler augmentation. An analysis of these layers allowed for targeted recommendations regarding filler placement to minimize complications and improve aesthetic outcomes. Supraorbital hollowness correction was best achieved with injections within the preseptal or sub-orbicularis oculi fat pads, while pretarsal fullness augmentation was most effective when fillers were placed close to the lash line to achieve the desired “charming roll” effect. The use of cannulas rather than needles in areas near critical vascular structures, such as the supratrochlear and supraorbital arteries, was emphasized to enhance safety by reducing the risk of vascular complications.

Through a comprehensive review of recent studies on filler applications in East Asian populations, we found that cultural aesthetic preferences significantly impact the preferred filler techniques, particularly for pretarsal fullness, commonly known as aegyo-sal among Koreans. Pretarsal augmentation was found to be particularly popular, as it enhances the youthful appearance of the eyes without altering natural contours. Additionally, a comparison of synthetic fillers with autologous fat transfer revealed that, while both approaches are effective, hyaluronic acid fillers provide a less invasive and reversible option with minimal recovery time, while fat transfer offers a more durable result for patients seeking a long-term correction of volume loss.

## 6. Discussion

The correction of supraorbital hollowness and enhancement of pretarsal fullness (commonly known as aegyo-sal) requires precision, particularly when addressing these areas in East Asian populations where specific anatomical features, such as reduced attachment of the levator muscle and unique fat distribution, impact aesthetic outcomes. Hyaluronic acid (HA) fillers are widely used for these enhancements because they provide a controlled, minimally invasive way to add volume, are reversible with hyaluronidase, and are associated with minimal recovery time. When injected at appropriate anatomical planes—particularly within the preseptal and sub-orbicularis fat pads—HA fillers can correct supraorbital hollowness while avoiding the complications often associated with superficial injections or intra-muscular placement, such as bruising or migration of the filler. In addition to synthetic fillers, autologous fat transfer, or lipofilling, serves as an effective alternative for volume restoration. Lipofilling utilizes the patient’s own fat, harvested through liposuction from areas such as the abdomen or thighs, and reinjected into the target site after processing. This technique offers distinct advantages, particularly for patients with pronounced volume deficits or those seeking a more permanent result. Fat transfer is associated with natural-looking, long-lasting effects due to the potential for the transferred fat cells to integrate and persist in the new site.

The study of Julieta et at. reviewed supraorbital anatomy, etiology, and pathophysiology, introducing a new classification for sunken upper eyelid and a low-risk filler technique using high-cohesivity hyaluronic acid. Thirty-two adults, predominantly female and without prior fillers, received HA injections in a two- or three-visit protocol, with results assessed across a year. Patients showed natural results without significant swelling, and only one required correction with hyaluronidase. Overall, the long-lasting and safe results suggest that cohesive HA is an effective, patient-satisfying option for non-surgical SUE treatment [1].

Supraorbital hollowness and flat eyebrows are interrelated conditions that significantly impact facial aesthetics, especially in East Asian populations. Supraorbital hollowness, characterized by a sunken appearance of the upper eyelid, is primarily influenced by genetic factors, aging, and sometimes surgical interventions such as blepharoplasty. The natural loss of fat in the orbital region, combined with anatomical variations like weaker or absent connections of the levator palpebrae superioris muscle to the dermal tissue, contributes to the sunken, tired look often seen in older adults [2,3,45,46,47,48,49,50]. This condition is further accentuated by the descent of subcutaneous fat and changes in the surrounding eyebrow region, creating a compound effect that alters the appearance of the entire periorbital area. Flat or drooping eyebrows exacerbate this hollow look by creating an unbalanced appearance that draws attention to the upper eyelid, emphasizing the signs of aging or fatigue [16,17].

The treatment of supraorbital hollowness requires a precise and careful approach to restore volume and achieve a more youthful contour. Injecting hyaluronic acid (HA) fillers into the appropriate layers of tissue can significantly improve the sunken appearance of the upper eyelid. However, the selection of the correct injection plane is critical to avoid complications such as hemorrhage, hematoma formation, or vascular compromise. Using a cannula instead of a needle can reduce the risk of intra-arterial injection, particularly around critical vascular structures like the supratrochlear and supraorbital arteries. Enhancing the orbital rim’s hollow areas can soften the prominent supratarsal lid crease and improve the overall eyelid contour, making the eyes appear more alert and rejuvenated. Nevertheless, achieving optimal results necessitates a comprehensive understanding of the anatomical variations and potential risks involved in the treatment of this delicate area [51].

Addressing flat eyebrows involves not just volumizing but also reshaping to restore their natural arch and position, which plays a pivotal role in overall facial aesthetics. The position and shape of the eyebrows influence the perception of youthfulness and emotional expressiveness. With age, the volume loss in the retro-orbicularis oculi fat (ROOF) and surrounding areas causes the eyebrows to sag, further contributing to an aged appearance [52,53,54,55]. By restoring volume to the eyebrow region, particularly lateral to the midpupillary line, and refining the shape through careful filler placement, practitioners can lift the eyebrows and enhance their natural contours. This approach not only improves the appearance of the eyebrows but also complements the correction of supraorbital hollowness, providing a balanced and harmonious rejuvenation of the upper face. A well-considered treatment plan that addresses both supraorbital hollowness and flat eyebrows can significantly enhance the patient’s overall appearance, creating a more vibrant and youthful look.

## Figures and Tables

**Table 1:** Summary of techniques for supraorbital hollowness and pretarsal fullness (aegyo-sal) treatments.

Treatment Area	Technique	Details	Safety Considerations	Amount
Supraorbital Hollowness	Retrograde linear threading and slow injection using small-particle HA filler	Patient in upright position, injections within preseptal and sub-orbicularis fat pads; maintains natural eyelid contour	Avoid injecting within orbicularis oculi muscle; use cannula near critical vascular structures to prevent complications	0.2–1 cc
Flat Eyebrows	Submuscular injection into ROOF (retro-orbicularis oculi fat) using a cannula	Focus on the area lateral to midpupillary line; soft filler injected to smooth surface	Avoid injecting near central retinal artery connections; ensure consistent depth	0.2–1.5 cc
Pretarsal Fullness (Aegyo-sal)	Dual-plane technique with filler injection beneath and within the orbicularis oculi muscle	Positioned close to the lower eyelashes, gradual injection into sections (central, lateral, and medial)	Avoid overly superficial injection to prevent lumps; maintain consistent depth to avoid vascular injury	0.3–1 cc

## Supplementary Materials

The following supporting information can be downloaded at: https://www.mdpi.com/article/10.3390/life15020304/s1, Video S1: Anatomy-Based Filler Injection: Treatment Techniques for Charming Roll.
